# Serological evidence of influenza D virus circulation among cattle in Poland

**DOI:** 10.2478/jvetres-2025-0048

**Published:** 2025-09-09

**Authors:** Małgorzata Kwaśnik, Jerzy Rola, Magdalena Larska, Wojciech Rożek

**Affiliations:** Department of Virology and Viral Animal Diseases, National Veterinary Research Institute, 24-100 Puławy, Poland

**Keywords:** cattle, haemagglutination, IDV, seroprevalence, virus neutralisation

## Abstract

**Introduction:**

The circulation of influenza D virus (IDV) has been confirmed in Europe, North America, Asia and Africa through seroprevalence and molecular studies, as well as by virus isolation. Its broad host range raises concerns about zoonotic potential, with cattle recognised as the natural reservoir. This study finds the seroprevalence of IDV in cattle, associates animal-level variables such as age, sex and origin and assesses the frequency of IDV exposure in small ruminants and horses in Poland.

**Material and Methods:**

A total of 1,029 serum samples were analysed, comprising 755 from cattle, 224 from small ruminants and 50 from horses. To assess the serological response, haemagglutination inhibition tests were performed, and samples classified as positive were subsequently tested with a virus neutralisation test.

**Results:**

The findings suggest possible circulation of IDV among cattle in Poland and give an estimated seroprevalence of 45.2%. Seroprevalence was related to age, with older cattle being more likely to test IDV seropositive. Regional differences were also observed, with the highest seroprevalence found in the Wielkopolskie voivodeship, an area known for its intensive cattle farming and one of the largest bovine populations in Poland, exceeding one million animals. No confirmation of IDV occurrence was obtained in any of the 274 sheep, goats, or horses.

**Conclusion:**

To the best of our knowledge, this is the first evidence of IDV circulation in the country, highlighting the need for further research to better understand the virus’ transmission pathways.

## Introduction

Influenza D virus (IDV) was first identified in 2011, following its isolation from pigs in the USA (6). However, cattle are considered the primary reservoir of the virus, which was first identified in the species in a retrospective study re-examining samples from 2003 (11). High seroprevalence of IDV was reported in cattle in Europe, Asia and the USA, and in some regions, the exposure exceeded 80% (14, 16, 22, 23, 27). Other animals susceptible to IDV infection include camels, pigs, small ruminants, dogs and horses, although their seroprevalence is lower than that observed in cattle (4, 13, 16, 20, 25). Among wild and captive animals, IDV infections have been confirmed in feral pigs, wild boars, deer, hedgehogs, giraffes, wildebeests, kangaroos, wallabies and llamas (2, 4, 5, 12, 15). Antibodies against IDV have also been detected in humans, particularly among individuals working with cattle (10). These findings suggest that influenza D may pose a zoonotic risk; therefore, the monitoring of the prevalence of IDV in both human and animals is expedient.

This virus primarily causes mild respiratory illness in cattle and upper respiratory tract lesions, but can also spread to the lower respiratory tract, potentially leading to pneumonia (21). However, the full spectrum of its clinical effects remains to be fully characterised. While IDV is less pathogenic than influenza A virus, it can still induce significant respiratory disorders, particularly in immunocompromised animals. Additionally, IDV may contribute to bovine respiratory disease (BRD) by influencing its onset and progression (19). The study aims to estimate the seroprevalence of IDV in cattle, determine the potential association between animal-level variables such as age, sex and origin, and assess what the frequency of IDV exposure is in small ruminants and horses in Poland.

## Material and Methods

### Viral strain and animal sera

Influenza D/sw/It/199724/3/2015 virus and influenza D hyperimmune serum with a titre of 2,560 were kindly provided by Dr Chiara Chiapponi (IZSLER, Brescia, Italy). A total of 755 sera from cattle, 224 sera from small ruminants and 50 sera from horses were used in the study. Sera from cattle and small ruminants were collected during bluetongue virus monitoring in Poland between 2022 and 2023 from four voivodeships: Dolnośląskie, Lubelskie, Podkarpackie and Wielkopolskie. Sample sizes were scaled to the cattle populations of the included voivodeships. Serum samples were also obtained from horses at a purebred Arabian stud farm. Available data included the age and sex of the animal and the year of sampling, but no information regarding animal health status was provided. No signs of respiratory disease or similar clinical issues were reported from the studied farms during the sampling period.

### Haemagglutination inhibition assay

Sera were treated with receptor-destroying enzyme RDE II (Denka Seiken Co., Ltd., Japan) at a 1 : 5 ratio for 12 h at 37°C, and were then heat inactivated for 1 h at 56°C. The haemagglutination inhibition (HI) assay was performed in duplicate using 0.5% chicken erythrocytes following the protocol outlined previously (7). Sera with HI titre ≥ 8 were classified as positive.

### Virus neutralisation test

Sera with HI titres ≥ 8 were subsequently tested using a virus neutralisation test (VNT), applying the method previously described and carrying it out in duplicate (24). Sera were heat inactivated at 56°C for 30 min and then diluted 1 : 2 with Eagle’s minimum essential medium supplemented with 0.5% foetal bovine serum and mixed with an equal volume of virus (100 TCID50/well) in a 96-well plate. After 1 h the mixture was added to Madin–Darby bovine kidney cells (1.5 × 10^5^ cells) in the 96-well plate. The plates were incubated at 37°C in 5% CO_2_. Haemagglutination activity in the supernatant was assessed after three days of incubation. The highest dilution of the serum that inactivated the virus (negative HA) was considered the virus neutralisation (VN) titre.

### Statistical analysis

The binomial results of the HI assay were presented as seroprevalence, which is the percentage of seropositive animals among the total animals tested or within a specific category of them. The 95% confidence intervals (95% CI) for the IDV seroprevalence, HI and VN titre results were calculated in total and in categories using the Clopper–Pearson exact method (1). The chi-squared test was applied to determine whether a statistically significant association existed between the age, sex or place of origin of the animals and IDV seroprevalence. The Spearman test was used to assess the association between the HI and VN titres, while the non-parametric Shapiro–Wilk and Mann–Whitney U tests were applied to examine the associations between HI titre and cattle age and voivodeship of origin and between VN titre and these variables. On the basis of the 25^th^ and 75^th^ percentiles, the cattle were categorised into three age groups: calves (≤1 year), young adults (2–4 years old) and older adults (≥5 years old). Statistical analysis was performed using Statistica v12.5 (PL) (Dell, Round Rock, TX, USA) and STATA v. 13.0 software (StataCorp, College Station, TX, USA). A P-value ≤ 0.05 was considered significant in all analyses.

## Results

**Fig. 1.**
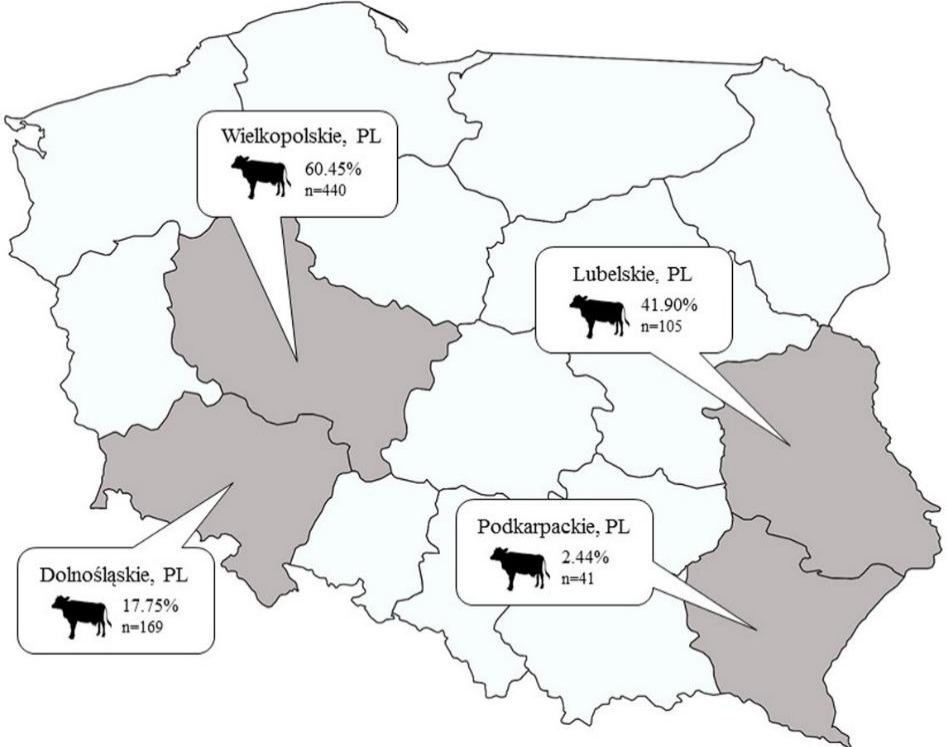
Voivodeship map of Poland. Grey shading indicates the regions of origin of cattle tested for anti–influenza D virus antibodies. The number of cattle tested and corresponding seroprevalence are provided for each region

Antibodies against IDV were detected in 341 out of 755 bovine sera (45.2%; 95% CI 41.6–48.8) using HI (Fig. 1).

**Fig. 2.**
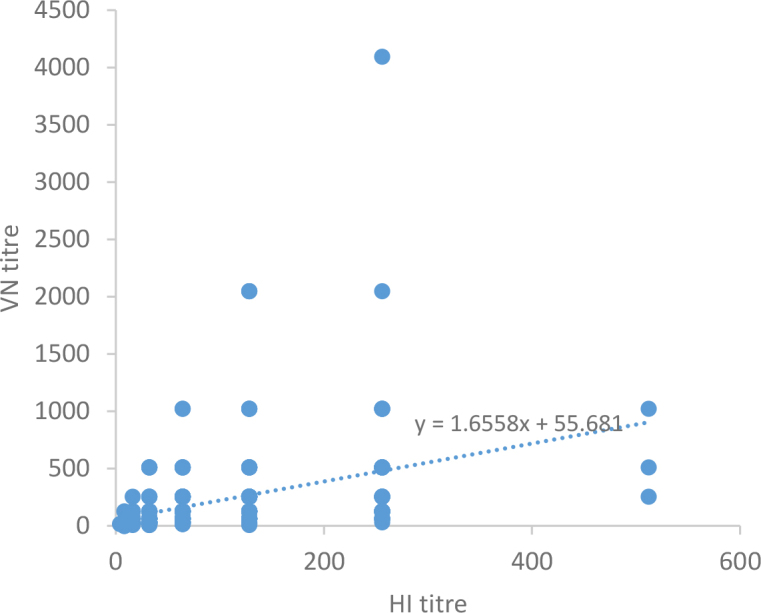
Scatter plots of influenza D virus antibody titres obtained by haemagglutination inhibition (HI) assay and virus neutralisation (VN) test. The positive slope (1.6558) suggests that an increase in HI titre was associated with a corresponding increase in VN titre

**Fig. 3.**
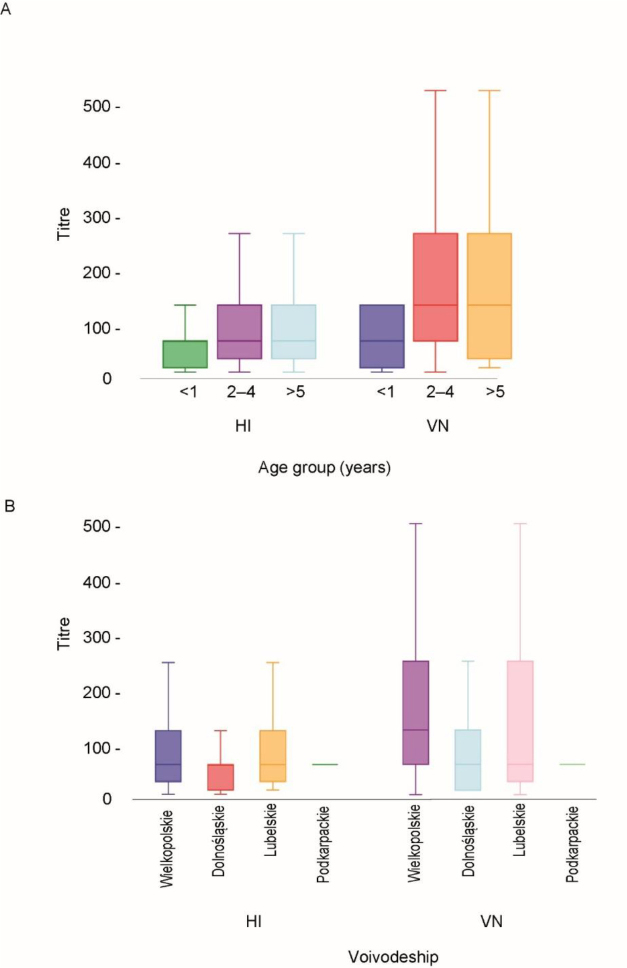
Box plot diagrams of the distribution of haemagglutinin inhibition (HI) and virus neutralisation (VN) titres in three age groups of cattle (A) and with regard to the origin of the animals tested (B); only positive HI and VN titres were considered. The whiskers are the 25^th^ and 75^th^ quartiles. The lines across the middle of the boxes identify the median values of titres

The HI titres ranged from 8 to 512, with a mean of 92.6 (95% CI 91.6–93.6). The VNT results were highly consistent with those of the HI assay, showing an agreement of 99.4%. Two samples had HI titres of 8 and tested negative in the VNT, leading to a marginal reduction in the observed seroprevalence of IDV-neutralising antibodies, which was calculated at 44.9% (95% CI: 41.3–48.5). The data on HI and VN titres, sex and voivodeship of origin of the sampled animals are presented in Supplementary Table S1. A significant association was observed between the HI and VN titres, with a Spearman’s ρ of 0.5739 (P-value < 0.0001) indicating a moderate positive correlation between the two. However, the median HI titre was significantly lower (P < 0.001) (64; 95% CI 16–256) than the median VN titre (128; 95% CI 16–512). The relationship between the HI and VNT results are shown in Fig. 2. Data insufficiency prevented the effect of sex on seroprevalence from being evaluated. Adult cattle showed significantly higher IDV seroprevalence than calves. Statistically significant differences in HI and VN titres between age groups were observed using the Mann–Whitney U test (P-value < 0.05). For HI titres, the z-scores were 6.85 for individuals aged 1 year or less compared to those aged 2–4 years and 5.42 for animals ≤1 year old compared to cattle ≥5 years old. For VN titres, the respective z-scores were 6.63 and 5.45. Seroprevalence varied by the region of animal origin, with the highest levels observed in the Wielkopolskie voivodeship, which accounted for the majority of samples. The HI and VN titres were also higher in samples from the Wielkopolskie voivodeship than in those from other regions (Mann–Whitney U test, P-value < 0.05). For HI titres, the z-scores were 8.72, 3.01 and 6.16 when comparing the Wielkopolskie with Dolnośląskie, Lubelskie and Podkarpackie voivodeships, respectively. The corresponding z-scores for VN titres were 8.77, 3.36 and 6.15. The distribution of HI and VN titres (considering positive values only) in relation to age groups and the voivodeship of origin of the tested animals are shown in Fig. 3.

The seroprevalence of IDV ranged from 2.4% to 60.4% and was associated with the age and voivodeship of origin of the cattle (Table 1).

**Table 1.** Analysis of influenza D virus seroprevalence in relation to cattle age, sex and voivodeship of origin

Variable	Seroprevalence in cattle in haemagglutination inhibition assay			
	n/N^1^	% (95% CI^2^)	Chi-squared	P-value
*Age group*				
Calves (≤1 year old)	11/98	11.2 (5.7–19.2)	55.2	<0.001
young adults 2–4 years old)	202/381	53.0 (47.9–58.1)		
older adults (≥5 years old)	128/276	46.4 (40.4–52.4)		
*Sex*				
female	71/283	25.1 (20.1–30.6)	2.5	<0.1
male	4/32	12.5 (3.5–29.0)		
missing data	266/440	60.4 (55.7–65.0)		
*Voivodeship*				
Dolnośląskie	30/169	17.8 (12.3–24.3)	123.5	<0.001
Lubelskie	44/105	41.9 (32.2–51.9)		
Podkarpackie	1/41	2.4 (0.1–12.8)		
Wielkopolskie	266/440	60.4 (55.7–65.0)		

1 – number of seropositive cattle/total number tested;

2 – binomial exact 95% confidence interval

None of the serum samples collected from sheep, goats or horses showed a positive result in the HI tests.

## Discussion

The circulation of IDV has been reported in various livestock and wildlife species across Europe and globally (8). Trends have emerged globally indicating a high prevalence of the virus in bovine populations, and a regional component appears to be manifested in Poland, indicated by the 45.2% seroprevalence of IDV in cattle found in the present research. To our knowledge, this study is the first to describe the seroprevalence of IDV in cattle in Poland. Several countries in Europe, including Ireland, Italy and Luxembourg, have reported seroprevalence rates exceeding 80% (14, 18, 23). These findings suggest that IDV circulates in cattle in these regions and can be considered hyperenzootic. Two of these hyperenzootic regions have yielded data pointing to a relationship between animal age and seropositivity. A study in Luxembourg found age to be a key factor for higher IDV seroprevalence, older beef cattle carrying IDV antibodies more frequently than younger dairy cattle, likely because the older cows had greater cumulative exposure to the virus (23). Similarly, in Ireland, lower seroprevalence was observed in cattle sampled for BRD diagnosis, which were typically younger animals than the Irish beef cattle sampled at slaughter and more frequently seropositive (14). In our study, adult cattle (the groups aged 2–4 years and older than 5 years) also showed significantly higher IDV seroprevalence compared to calves (the group aged 1 year or less). Instances of potential exposure accumulate over time: the older the animal, the greater the chance of infection, especially where no immunisation is implemented. In such cases, the strength of population immunity will depend on the frequency of instances of potential exposure to the circulating virus. By inference, we can also suspect that bovine influenza D is not a calving-age disease, unlike bovine respiratory syncytial virus, where exposure in calves can be higher and can result in the development of BRD frequently in this age group. However, it is important to note that seroprevalence alone does not indicate the likelihood of symptomatic disease having followed IDV infection.

A study conducted in France reported an average IDV seroprevalence of 47.2% and it analysed data by region to highlight the significance of breeding systems (16). The highest seroprevalence was found in regions with a high density of fattening units for young bulls and calves, where frequent animal exchanges between farms and the introduction of livestock from diverse origins likely facilitated the spread of the virus. Conversely, regions with lower seroprevalence were predominantly characterised by traditional dairy and beef cattle farming systems. In our study, the highest seroprevalence was observed for cattle in the Wielkopolskie voivodeship, which is noted for intensive cattle production, with over a million animals and one of the largest cattle populations in the country (17). In the Lubelskie voivodeship, where the cattle population is close to the national average, the seroprevalence (41.9%) was similar to the average of this study (45.2%). The low IDV seroprevalence in the Podkarpackie voivodeship, characterised by a proportionately smaller cattle population, may be attributed to the low number of cattle tested. Therefore, reaching a definitive conclusion that IDV exposure in Poland is related to the size and density of the cattle population requires further research with a representative number of samples from each voivodeship.

The high seroprevalence of IDV in cattle can also be attributed to the subclinical nature of the infection, which allows cattle to develop immunity without showing clear clinical symptoms. Additionally, Wan *et al*. (26) suggested that, alongside epidemiological factors, the ineffectiveness of existing immunity, which they demonstrated with experimental reinfection of cattle, and the cocirculation of diverse viral strains contribute to IDV’s high prevalence in animal populations. Gaudino *et al*. (3), analysing the distribution of IDV in Europe, hypothesised that livestock trade between European countries may facilitate the spread of the virus, thus highlighting the importance of both local management and global cattle movement practices in influencing the spread of IDV.

There are far fewer data on IDV seroprevalence in small ruminants and horses compared to cattle. Overall, the observed seroprevalence is generally lower than in cattle, camels or pigs. In France, a study showed seroprevalence of 0.5% in sheep and 3.2% in goats (16), and research in Ireland found 4.5% in sheep (14). However, recent studies from Italy confirmed considerably higher seroprevalence, which had been reported before. In Sicily, out of 600 ovine serum samples tested, 168 (28.0%) were positive for IDV D/660 or D/OK or both by the HI method, while 378 (63.0%) of the samples were positive for IDV D/660 or D/OK or both by the VN method (9). In our study, IDV antibodies were absent from all goat, sheep and horse sera examined.

In this study, both HI and VN assays were employed to improve the reliability of serological detection of IDV. While HI is widely regarded as the gold standard in influenza surveillance because of its specificity, it may yield false-positive or false-negative results. The addition of the VN test, known for its higher sensitivity and functional relevance, allowed for cross-validation of results, helping to address potential inconsistencies and increasing confidence in the findings.

Our study acknowledges some limitations that may have contributed to some underestimation in the obtained results. Specifically, we used an IDV strain representing only one of the two dominant phylogenetic lineages in Europe, D/OK, for the HI and VNT. Furthermore, the sample size for small ruminants and horses was relatively small. Another limitation is the lack of information regarding the health status or medical history of the sampled animals. However, no enrolled farm notified us of any signs of respiratory disease or similar clinical issues during the sampling period.

## Conclusion

This study suggests the possible circulation of IDV among cattle in Poland and estimates its seroprevalence at 45.2%. This level is comparable to those reported in other European countries. Analysis of the age groups of the cattle studied showed significantly higher IDV seroprevalence in older animals than in calves. No antibodies to IDV were detected in the small ruminants or horses. Based on the findings from our country and others, it can be assumed that age, the breeding system, national and international transportation of animals and the specificity of the virus itself may influence seroprevalence and cause the high seropositivity found in cattle in Europe and worldwide. Given IDV’s potential for variability through antigenic drift and reassortment, and its wide host range across multiple livestock species, controlling its spread poses significant challenges.

## Supplementary Material

Supplementary Material Details
