# Supplementary material for: Serological evidence of influenza D virus circulation among cattle in Poland
Source: J Vet Res. 2025 Sep 9;69(3):305–11. doi: 10.2478/jvetres-2025-0048 (PMC12503218; doi:10.2478/jvetres-2025-0048)
Supplement: Supplementary file 1 — Supplementary Material Details [file jvetres-2025-0048_sm.pdf]

Supplementary Table S1. Data on sampled animals, including age, sex, region of origin, and serological test results for IDV based on hemagglutination inhibition (HI) and virus neutralization (VN) tests

Sample	HI	VN	Age M	Sex	Voivodeship
1.	32	64	36	N/A	Wielkopolskie
2.	32	32	36	N/A	Wielkopolskie
3.	128	64	36	N/A	Wielkopolskie
4.	64	128	48	N/A	Wielkopolskie
5.	64	512	36	N/A	Wielkopolskie
6.	128	128	36	N/A	Wielkopolskie
7.	128	256	48	N/A	Wielkopolskie
8.	64	64	24	N/A	Wielkopolskie
9.	32	512	24	N/A	Wielkopolskie
10.	128	1024	72	N/A	Wielkopolskie
11.	32	64	60	N/A	Wielkopolskie
12.	32	32	36	N/A	Wielkopolskie
13.	32	128	36	N/A	Wielkopolskie
14.	64	64	60	N/A	Wielkopolskie
15.	128	64	36	N/A	Wielkopolskie
16.	256	256	108	N/A	Wielkopolskie
17.	128	512	36	N/A	Wielkopolskie
18.	128	2048	48	N/A	Wielkopolskie
19.	128	64	48	N/A	Wielkopolskie
20.	64	256	24	N/A	Wielkopolskie
21.	128	2048	24	N/A	Wielkopolskie
22.	128	64	24	N/A	Wielkopolskie
23.	64	1024	49	N/A	Wielkopolskie
24.	64	128	60	N/A	Wielkopolskie
25.	64	64	5	N/A	Wielkopolskie
26.	64	64	36	N/A	Wielkopolskie
27.	128	256	48	N/A	Wielkopolskie
28.	128	1024	36	N/A	Wielkopolskie
29.	64	128	24	N/A	Wielkopolskie
30.	64	256	60	N/A	Wielkopolskie
31.	64	1024	24	N/A	Wielkopolskie
32.	64	128	60	N/A	Wielkopolskie
33.	64	128	48	N/A	Wielkopolskie
34.	8	0	48	N/A	Wielkopolskie
35.	128	64	48	N/A	Wielkopolskie
36.	128	512	48	N/A	Wielkopolskie
37.	16	32	36	N/A	Wielkopolskie
38.	32	256	48	N/A	Wielkopolskie
39.	64	64	36	N/A	Wielkopolskie
40.	128	2048	60	N/A	Wielkopolskie
41.	32	16	48	N/A	Wielkopolskie
42.	256	512	48	N/A	Wielkopolskie
43.	64	128	48	N/A	Wielkopolskie

44.	256	512	84	N/A	Wielkopolskie
45.	256	64	84	N/A	Wielkopolskie
46.	128	32	48	N/A	Wielkopolskie
47.	64	512	84	N/A	Wielkopolskie
48.	32	512	24	N/A	Wielkopolskie
49.	256	512	48	N/A	Wielkopolskie
50.	128	256	72	N/A	Wielkopolskie
51.	256	512	48	N/A	Wielkopolskie
52.	32	64	36	N/A	Wielkopolskie
53.	16	64	36	N/A	Wielkopolskie
54.	32	512	36	N/A	Wielkopolskie
55.	64	256	60	N/A	Wielkopolskie
56.	128	128	36	N/A	Wielkopolskie
57.	256	4096	60	N/A	Wielkopolskie
58.	32	64	24	N/A	Wielkopolskie
59.	256	64	36	N/A	Wielkopolskie
60.	64	128	36	N/A	Wielkopolskie
61.	32	512	108	N/A	Wielkopolskie
62.	64	512	36	N/A	Wielkopolskie
63.	16	256	24	N/A	Wielkopolskie
64.	16	64	24	N/A	Wielkopolskie
65.	256	64	24	N/A	Wielkopolskie
66.	256	512	24	N/A	Wielkopolskie
67.	128	256	24	N/A	Wielkopolskie
68.	128	256	24	N/A	Wielkopolskie
69.	256	512	108	N/A	Wielkopolskie
70.	64	256	36	N/A	Wielkopolskie
71.	64	128	72	N/A	Wielkopolskie
72.	32	128	108	N/A	Wielkopolskie
73.	64	128	96	N/A	Wielkopolskie
74.	32	128	36	N/A	Wielkopolskie
75.	64	128	36	N/A	Wielkopolskie
76.	32	16	24	N/A	Wielkopolskie
77.	32	64	24	N/A	Wielkopolskie
78.	16	64	36	N/A	Wielkopolskie
79.	128	128	12	N/A	Wielkopolskie
80.	128	64	48	N/A	Wielkopolskie
81.	256	128	72	N/A	Wielkopolskie
82.	256	256	60	N/A	Wielkopolskie
83.	16	16	24	N/A	Wielkopolskie
84.	16	16	12	N/A	Wielkopolskie
85.	128	16	48	N/A	Wielkopolskie
86.	64	256	24	N/A	Wielkopolskie
87.	128	256	48	N/A	Wielkopolskie
88.	16	128	12	N/A	Wielkopolskie
89.	256	512	72	N/A	Wielkopolskie

90.	16	16	24	N/A	Wielkopolskie
91.	256	64	120	N/A	Wielkopolskie
92.	64	64	24	N/A	Wielkopolskie
93.	64	128	48	N/A	Wielkopolskie
94.	128	128	36	N/A	Wielkopolskie
95.	128	256	36	N/A	Wielkopolskie
96.	64	64	48	N/A	Wielkopolskie
97.	128	256	60	N/A	Wielkopolskie
98.	32	8	48	N/A	Wielkopolskie
99.	128	128	48	N/A	Wielkopolskie
100.	256	512	60	N/A	Wielkopolskie
101.	64	128	24	N/A	Wielkopolskie
102.	64	64	24	N/A	Wielkopolskie
103.	64	32	108	N/A	Wielkopolskie
104.	16	128	24	N/A	Wielkopolskie
105.	128	256	24	N/A	Wielkopolskie
106.	64	256	36	N/A	Wielkopolskie
107.	64	128	48	N/A	Wielkopolskie
108.	128	512	24	N/A	Wielkopolskie
109.	32	64	24	N/A	Wielkopolskie
110.	256	128	24	N/A	Wielkopolskie
111.	32	64	24	N/A	Wielkopolskie
112.	16	16	24	N/A	Wielkopolskie
113.	64	16	24	N/A	Wielkopolskie
114.	128	64	24	N/A	Wielkopolskie
115.	256	256	24	N/A	Wielkopolskie
116.	16	16	24	N/A	Wielkopolskie
117.	32	128	24	N/A	Wielkopolskie
118.	64	64	24	N/A	Wielkopolskie
119.	32	32	24	N/A	Wielkopolskie
120.	64	64	24	N/A	Wielkopolskie
121.	256	128	24	N/A	Wielkopolskie
122.	16	64	24	N/A	Wielkopolskie
123.	128	64	24	N/A	Wielkopolskie
124.	128	256	24	N/A	Wielkopolskie
125.	64	64	24	N/A	Wielkopolskie
126.	32	64	24	N/A	Wielkopolskie
127.	256	256	24	N/A	Wielkopolskie
128.	32	64	24	N/A	Wielkopolskie
129.	64	32	24	N/A	Wielkopolskie
130.	128	64	24	N/A	Wielkopolskie
131.	32	32	24	N/A	Wielkopolskie
132.	64	64	24	N/A	Wielkopolskie
133.	128	256	24	N/A	Wielkopolskie
134.	64	32	24	N/A	Wielkopolskie
135.	64	64	24	N/A	Wielkopolskie

136.	32	64	48	N/A	Wielkopolskie
137.	64	128	36	N/A	Wielkopolskie
138.	64	32	36	N/A	Wielkopolskie
139.	16	8	12	N/A	Wielkopolskie
140.	32	64	24	N/A	Wielkopolskie
141.	128	128	48	N/A	Wielkopolskie
142.	32	64	24	N/A	Wielkopolskie
143.	16	32	36	N/A	Wielkopolskie
144.	8	32	84	N/A	Wielkopolskie
145.	8	32	72	N/A	Wielkopolskie
146.	16	32	96	N/A	Wielkopolskie
147.	16	64	72	N/A	Wielkopolskie
148.	8	16	60	N/A	Wielkopolskie
149.	32	64	120	N/A	Wielkopolskie
150.	16	128	24	N/A	Wielkopolskie
151.	8	32	24	N/A	Wielkopolskie
152.	8	32	24	N/A	Wielkopolskie
153.	128	128	60	N/A	Wielkopolskie
154.	128	256	36	N/A	Wielkopolskie
155.	32	32	24	N/A	Wielkopolskie
156.	16	32	24	N/A	Wielkopolskie
157.	128	512	36	N/A	Wielkopolskie
158.	128	256	36	N/A	Wielkopolskie
159.	128	64	48	N/A	Wielkopolskie
160.	64	256	72	N/A	Wielkopolskie
161.	128	256	48	N/A	Wielkopolskie
162.	16	16	48	N/A	Wielkopolskie
163.	32	16	48	N/A	Wielkopolskie
164.	16	16	24	N/A	Wielkopolskie
165.	32	128	48	N/A	Wielkopolskie
166.	128	32	72	N/A	Wielkopolskie
167.	32	16	120	N/A	Wielkopolskie
168.	32	64	72	N/A	Wielkopolskie
169.	128	128	60	N/A	Wielkopolskie
170.	128	256	60	N/A	Wielkopolskie
171.	32	64	96	N/A	Wielkopolskie
172.	128	256	60	N/A	Wielkopolskie
173.	64	16	60	N/A	Wielkopolskie
174.	64	16	24	N/A	Wielkopolskie
175.	32	32	72	N/A	Wielkopolskie
176.	64	64	84	N/A	Wielkopolskie
177.	64	128	60	N/A	Wielkopolskie
178.	32	32	24	N/A	Wielkopolskie
179.	64	256	72	N/A	Wielkopolskie
180.	32	256	48	N/A	Wielkopolskie
181.	128	128	12	N/A	Wielkopolskie

182.	64	32	24	N/A	Wielkopolskie
183.	64	128	132	N/A	Wielkopolskie
184.	128	128	48	N/A	Wielkopolskie
185.	128	128	108	N/A	Wielkopolskie
186.	128	512	72	N/A	Wielkopolskie
187.	128	64	84	N/A	Wielkopolskie
188.	128	512	120	N/A	Wielkopolskie
189.	256	128	108	N/A	Wielkopolskie
190.	64	64	120	N/A	Wielkopolskie
191.	256	128	48	N/A	Wielkopolskie
192.	256	32	96	N/A	Wielkopolskie
193.	16	32	24	N/A	Wielkopolskie
194.	32	256	96	N/A	Wielkopolskie
195.	256	1024	84	N/A	Wielkopolskie
196.	128	512	96	N/A	Wielkopolskie
197.	128	128	60	N/A	Wielkopolskie
198.	128	64	96	N/A	Wielkopolskie
199.	256	1024	48	N/A	Wielkopolskie
200.	128	256	48	N/A	Wielkopolskie
201.	256	2048	84	N/A	Wielkopolskie
202.	256	512	48	N/A	Wielkopolskie
203.	64	32	120	N/A	Wielkopolskie
204.	256	1024	36	N/A	Wielkopolskie
205.	128	128	36	N/A	Wielkopolskie
206.	32	64	36	N/A	Wielkopolskie
207.	512	512	84	N/A	Wielkopolskie
208.	64	512	48	N/A	Wielkopolskie
209.	64	512	24	N/A	Wielkopolskie
210.	128	128	36	N/A	Wielkopolskie
211.	64	256	36	N/A	Wielkopolskie
212.	32	128	60	N/A	Wielkopolskie
213.	64	32	36	N/A	Wielkopolskie
214.	32	32	24	N/A	Wielkopolskie
215.	32	32	36	N/A	Wielkopolskie
216.	64	256	48	N/A	Wielkopolskie
217.	32	128	36	N/A	Wielkopolskie
218.	32	128	72	N/A	Wielkopolskie
219.	32	256	36	N/A	Wielkopolskie
220.	128	512	60	N/A	Wielkopolskie
221.	64	64	60	N/A	Wielkopolskie
222.	128	512	48	N/A	Wielkopolskie
223.	64	64	84	N/A	Wielkopolskie
224.	128	64	84	N/A	Wielkopolskie
225.	64	32	72	N/A	Wielkopolskie
226.	256	1024	36	N/A	Wielkopolskie
227.	32	32	72	N/A	Wielkopolskie

228.	64	128	36	N/A	Wielkopolskie
229.	128	1024	36	N/A	Wielkopolskie
230.	64	32	36	N/A	Wielkopolskie
231.	128	256	36	N/A	Wielkopolskie
232.	64	64	24	N/A	Wielkopolskie
233.	128	128	24	N/A	Wielkopolskie
234.	128	128	36	N/A	Wielkopolskie
235.	128	64	36	N/A	Wielkopolskie
236.	64	128	36	N/A	Wielkopolskie
237.	256	128	36	N/A	Wielkopolskie
238.	64	128	24	N/A	Wielkopolskie
239.	64	256	24	N/A	Wielkopolskie
240.	32	16	24	N/A	Wielkopolskie
241.	32	64	24	N/A	Wielkopolskie
242.	32	32	36	N/A	Wielkopolskie
243.	32	128	48	N/A	Wielkopolskie
244.	64	128	24	N/A	Wielkopolskie
245.	64	64	48	N/A	Wielkopolskie
246.	64	128	60	N/A	Wielkopolskie
247.	64	128	12	N/A	Wielkopolskie
248.	8	32	24	N/A	Wielkopolskie
249.	8	16	96	N/A	Wielkopolskie
250.	64	128	60	N/A	Wielkopolskie
251.	64	64	12	N/A	Wielkopolskie
252.	8	0	24	N/A	Wielkopolskie
253.	64	256	36	N/A	Wielkopolskie
254.	64	64	12	N/A	Wielkopolskie
255.	128	128	24	N/A	Wielkopolskie
256.	128	128	36	N/A	Wielkopolskie
257.	128	256	48	N/A	Wielkopolskie
258.	256	512	24	N/A	Wielkopolskie
259.	256	512	36	N/A	Wielkopolskie
260.	256	256	48	N/A	Wielkopolskie
261.	64	128	48	N/A	Wielkopolskie
262.	64	64	180	N/A	Wielkopolskie
263.	32	64	120	N/A	Wielkopolskie
264.	128	1024	36	N/A	Wielkopolskie
265.	128	256	96	N/A	Wielkopolskie
266.	16	32	36	N/A	Wielkopolskie
267.	-	-	24	N/A	Wielkopolskie
268.	-	-	36	N/A	Wielkopolskie
269.	-	-	12	N/A	Wielkopolskie
270.	-	-	12	N/A	Wielkopolskie
271.	-	-	12	N/A	Wielkopolskie
272.	-	-	7	N/A	Wielkopolskie
273.	-	-	7	N/A	Wielkopolskie

274.	-	-	7	N/A	Wielkopolskie
275.	-	-	9	N/A	Wielkopolskie
276.	-	-	8	N/A	Wielkopolskie
277.	-	-	8	N/A	Wielkopolskie
278.	-	-	8	N/A	Wielkopolskie
279.	-	-	12	N/A	Wielkopolskie
280.	-	-	24	N/A	Wielkopolskie
281.	-	-	24	N/A	Wielkopolskie
282.	-	-	24	N/A	Wielkopolskie
283.	-	-	36	N/A	Wielkopolskie
284.	-	-	72	N/A	Wielkopolskie
285.	-	-	36	N/A	Wielkopolskie
286.	-	-	24	N/A	Wielkopolskie
287.	-	-	60	N/A	Wielkopolskie
288.	-	-	48	N/A	Wielkopolskie
289.	-	-	48	N/A	Wielkopolskie
290.	-	-	60	N/A	Wielkopolskie
291.	-	-	48	N/A	Wielkopolskie
292.	-	-	36	N/A	Wielkopolskie
293.	-	-	48	N/A	Wielkopolskie
294.	-	-	60	N/A	Wielkopolskie
295.	-	-	36	N/A	Wielkopolskie
296.	-	-	36	N/A	Wielkopolskie
297.	-	-	48	N/A	Wielkopolskie
298.	-	-	84	N/A	Wielkopolskie
299.	-	-	60	N/A	Wielkopolskie
300.	-	-	120	N/A	Wielkopolskie
301.	-	-	84	N/A	Wielkopolskie
302.	-	-	72	N/A	Wielkopolskie
303.	-	-	48	N/A	Wielkopolskie
304.	-	-	48	N/A	Wielkopolskie
305.	-	-	108	N/A	Wielkopolskie
306.	-	-	120	N/A	Wielkopolskie
307.	-	-	108	N/A	Wielkopolskie
308.	-	-	144	N/A	Wielkopolskie
309.	-	-	60	N/A	Wielkopolskie
310.	-	-	60	N/A	Wielkopolskie
311.	-	-	24	N/A	Wielkopolskie
312.	-	-	24	N/A	Wielkopolskie
313.	-	-	36	N/A	Wielkopolskie
314.	-	-	36	N/A	Wielkopolskie
315.	-	-	24	N/A	Wielkopolskie
316.	-	-	96	N/A	Wielkopolskie
317.	-	-	24	N/A	Wielkopolskie
318.	-	-	36	N/A	Wielkopolskie
319.	-	-	48	N/A	Wielkopolskie

320.	-	-	10	N/A	Wielkopolskie
321.	-	-	10	N/A	Wielkopolskie
322.	-	-	12	N/A	Wielkopolskie
323.	-	-	10	N/A	Wielkopolskie
324.	-	-	24	N/A	Wielkopolskie
325.	-	-	24	N/A	Wielkopolskie
326.	-	-	24	N/A	Wielkopolskie
327.	-	-	12	N/A	Wielkopolskie
328.	-	-	10	N/A	Wielkopolskie
329.	-	-	10	N/A	Wielkopolskie
330.	-	-	48	N/A	Wielkopolskie
331.	-	-	60	N/A	Wielkopolskie
332.	-	-	72	N/A	Wielkopolskie
333.	-	-	60	N/A	Wielkopolskie
334.	-	-	60	N/A	Wielkopolskie
335.	-	-	24	N/A	Wielkopolskie
336.	-	-	24	N/A	Wielkopolskie
337.	-	-	24	N/A	Wielkopolskie
338.	-	-	12	N/A	Wielkopolskie
339.	-	-	84	N/A	Wielkopolskie
340.	-	-	36	N/A	Wielkopolskie
341.	-	-	36	N/A	Wielkopolskie
342.	-	-	36	N/A	Wielkopolskie
343.	-	-	36	N/A	Wielkopolskie
344.	-	-	48	N/A	Wielkopolskie
345.	-	-	24	N/A	Wielkopolskie
346.	-	-	24	N/A	Wielkopolskie
347.	-	-	108	N/A	Wielkopolskie
348.	-	-	84	N/A	Wielkopolskie
349.	-	-	24	N/A	Wielkopolskie
350.	-	-	24	N/A	Wielkopolskie
351.	-	-	24	N/A	Wielkopolskie
352.	-	-	12	N/A	Wielkopolskie
353.	-	-	24	N/A	Wielkopolskie
354.	-	-	48	N/A	Wielkopolskie
355.	-	-	60	N/A	Wielkopolskie
356.	-	-	24	N/A	Wielkopolskie
357.	-	-	24	N/A	Wielkopolskie
358.	-	-	72	N/A	Wielkopolskie
359.	-	-	48	N/A	Wielkopolskie
360.	-	-	24	N/A	Wielkopolskie
361.	-	-	60	N/A	Wielkopolskie
362.	-	-	24	N/A	Wielkopolskie
363.	-	-	60	N/A	Wielkopolskie
364.	-	-	24	N/A	Wielkopolskie
365.	-	-	12	N/A	Wielkopolskie

366.	-	-	24	N/A	Wielkopolskie
367.	-	-	12	N/A	Wielkopolskie
368.	-	-	12	N/A	Wielkopolskie
369.	-	-	120	N/A	Wielkopolskie
370.	-	-	12	N/A	Wielkopolskie
371.	-	-	12	N/A	Wielkopolskie
372.	-	-	12	N/A	Wielkopolskie
373.	-	-	60	N/A	Wielkopolskie
374.	-	-	96	N/A	Wielkopolskie
375.	-	-	108	N/A	Wielkopolskie
376.	-	-	84	N/A	Wielkopolskie
377.	-	-	36	N/A	Wielkopolskie
378.	-	-	24	N/A	Wielkopolskie
379.	-	-	24	N/A	Wielkopolskie
380.	-	-	24	N/A	Wielkopolskie
381.	-	-	24	N/A	Wielkopolskie
382.	-	-	36	N/A	Wielkopolskie
383.	-	-	36	N/A	Wielkopolskie
384.	-	-	36	N/A	Wielkopolskie
385.	-	-	60	N/A	Wielkopolskie
386.	-	-	60	N/A	Wielkopolskie
387.	-	-	36	N/A	Wielkopolskie
388.	-	-	24	N/A	Wielkopolskie
389.	-	-	36	N/A	Wielkopolskie
390.	-	-	6	N/A	Wielkopolskie
391.	-	-	6	N/A	Wielkopolskie
392.	-	-	24	N/A	Wielkopolskie
393.	-	-	72	N/A	Wielkopolskie
394.	-	-	48	N/A	Wielkopolskie
395.	-	-	36	N/A	Wielkopolskie
396.	-	-	48	N/A	Wielkopolskie
397.	-	-	48	N/A	Wielkopolskie
398.	-	-	48	N/A	Wielkopolskie
399.	-	-	48	N/A	Wielkopolskie
400.	-	-	48	N/A	Wielkopolskie
401.	-	-	36	N/A	Wielkopolskie
402.	-	-	48	N/A	Wielkopolskie
403.	-	-	48	N/A	Wielkopolskie
404.	-	-	144	N/A	Wielkopolskie
405.	-	-	96	N/A	Wielkopolskie
406.	-	-	96	N/A	Wielkopolskie
407.	-	-	96	N/A	Wielkopolskie
408.	-	-	24	N/A	Wielkopolskie
409.	-	-	12	N/A	Wielkopolskie
410.	-	-	10	N/A	Wielkopolskie
411.	-	-	10	N/A	Wielkopolskie

412.	-	-	10	N/A	Wielkopolskie
413.	-	-	12	N/A	Wielkopolskie
414.	-	-	10	N/A	Wielkopolskie
415.	-	-	5	N/A	Wielkopolskie
416.	-	-	8	N/A	Wielkopolskie
417.	-	-	9	N/A	Wielkopolskie
418.	-	-	10	N/A	Wielkopolskie
419.	-	-	9	N/A	Wielkopolskie
420.	-	-	10	N/A	Wielkopolskie
421.	-	-	10	N/A	Wielkopolskie
422.	-	-	10	N/A	Wielkopolskie
423.	-	-	8	N/A	Wielkopolskie
424.	-	-	8	N/A	Wielkopolskie
425.	-	-	9	N/A	Wielkopolskie
426.	-	-	9	N/A	Wielkopolskie
427.	-	-	9	N/A	Wielkopolskie
428.	-	-	36	N/A	Wielkopolskie
429.	-	-	60	N/A	Wielkopolskie
430.	-	-	24	N/A	Wielkopolskie
431.	-	-	60	N/A	Wielkopolskie
432.	-	-	36	N/A	Wielkopolskie
433.	-	-	36	N/A	Wielkopolskie
434.	-	-	5	N/A	Wielkopolskie
435.	-	-	24	N/A	Wielkopolskie
436.	-	-	48	N/A	Wielkopolskie
437.	-	-	36	N/A	Wielkopolskie
438.	-	-	36	N/A	Wielkopolskie
439.	-	-	36	N/A	Wielkopolskie
440.	-	-	36	N/A	Wielkopolskie
441.	64	128	60	M	Dolnośląskie
442.	64	64	84	F	Dolnośląskie
443.	16	16	60	F	Dolnośląskie
444.	16	16	60	F	Dolnośląskie
445.	16	32	60	F	Dolnośląskie
446.	128	128	48	F	Dolnośląskie
447.	64	64	48	F	Dolnośląskie
448.	8	16	72	F	Dolnośląskie
449.	64	32	72	F	Dolnośląskie
450.	64	64	72	F	Dolnośląskie
451.	64	32	24	F	Dolnośląskie
452.	128	128	24	F	Dolnośląskie
453.	64	128	36	F	Dolnośląskie
454.	64	256	108	F	Dolnośląskie
455.	256	128	72	F	Dolnośląskie
456.	128	256	84	F	Dolnośląskie
457.	256	128	84	F	Dolnośląskie

458.	8	16	60	F	Dolnośląskie
459.	8	16	10	F	Dolnośląskie
460.	8	16	12	M	Dolnośląskie
461.	16	32	120	F	Dolnośląskie
462.	16	16	60	F	Dolnośląskie
463.	8	16	108	F	Dolnośląskie
464.	16	64	36	F	Dolnośląskie
465.	8	128	36	F	Dolnośląskie
466.	128	256	84	F	Dolnośląskie
467.	64	64	72	F	Dolnośląskie
468.	32	32	24	F	Dolnośląskie
469.	128	128	144	F	Dolnośląskie
470.	64	128	144	F	Dolnośląskie
471.	-	-	48	F	Dolnośląskie
472.	-	-	72	F	Dolnośląskie
473.	-	-	72	F	Dolnośląskie
474.	-	-	24	F	Dolnośląskie
475.	-	-	24	F	Dolnośląskie
476.	-	-	36	M	Dolnośląskie
477.	-	-	36	F	Dolnośląskie
478.	-	-	84	F	Dolnośląskie
479.	-	-	96	F	Dolnośląskie
480.	-	-	96	F	Dolnośląskie
481.	-	-	144	F	Dolnośląskie
482.	-	-	36	F	Dolnośląskie
483.	-	-	144	F	Dolnośląskie
484.	-	-	24	M	Dolnośląskie
485.	-	-	24	M	Dolnośląskie
486.	-	-	12	M	Dolnośląskie
487.	-	-	12	M	Dolnośląskie
488.	-	-	12	F	Dolnośląskie
489.	-	-	24	F	Dolnośląskie
490.	-	-	36	F	Dolnośląskie
491.	-	-	24	F	Dolnośląskie
492.	-	-	12	M	Dolnośląskie
493.	-	-	12	M	Dolnośląskie
494.	-	-	4	M	Dolnośląskie
495.	-	-	60	F	Dolnośląskie
496.	-	-	9	M	Dolnośląskie
497.	-	-	7	M	Dolnośląskie
498.	-	-	7	M	Dolnośląskie
499.	-	-	24	F	Dolnośląskie
500.	-	-	36	F	Dolnośląskie
501.	-	-	60	F	Dolnośląskie
502.	-	-	7	M	Dolnośląskie
503.	-	-	7	M	Dolnośląskie

504.	-	-	12	F	Dolnośląskie
505.	-	-	4	F	Dolnośląskie
506.	-	-	4	F	Dolnośląskie
507.	-	-	96	F	Dolnośląskie
508.	-	-	96	F	Dolnośląskie
509.	-	-	10	M	Dolnośląskie
510.	-	-	12	M	Dolnośląskie
511.	-	-	24	F	Dolnośląskie
512.	-	-	24	F	Dolnośląskie
513.	-	-	7	M	Dolnośląskie
514.	-	-	132	F	Dolnośląskie
515.	-	-	96	F	Dolnośląskie
516.	-	-	60	F	Dolnośląskie
517.	-	-	12	F	Dolnośląskie
518.	-	-	4	F	Dolnośląskie
519.	-	-	48	F	Dolnośląskie
520.	-	-	48	F	Dolnośląskie
521.	-	-	60	F	Dolnośląskie
522.	-	-	144	F	Dolnośląskie
523.	-	-	132	F	Dolnośląskie
524.	-	-	96	F	Dolnośląskie
525.	-	-	96	F	Dolnośląskie
526.	-	-	36	F	Dolnośląskie
527.	-	-	84	F	Dolnośląskie
528.	-	-	60	F	Dolnośląskie
529.	-	-	48	F	Dolnośląskie
530.	-	-	60	F	Dolnośląskie
531.	-	-	72	F	Dolnośląskie
532.	-	-	36	F	Dolnośląskie
533.	-	-	36	F	Dolnośląskie
534.	-	-	36	F	Dolnośląskie
535.	-	-	36	F	Dolnośląskie
536.	-	-	168	F	Dolnośląskie
537.	-	-	24	F	Dolnośląskie
538.	-	-	120	F	Dolnośląskie
539.	-	-	168	F	Dolnośląskie
540.	-	-	72	F	Dolnośląskie
541.	-	-	120	F	Dolnośląskie
542.	-	-	204	F	Dolnośląskie
543.	-	-	72	F	Dolnośląskie
544.	-	-	24	F	Dolnośląskie
545.	-	-	48	F	Dolnośląskie
546.	-	-	144	F	Dolnośląskie
547.	-	-	96	F	Dolnośląskie
548.	-	-	144	F	Dolnośląskie
549.	-	-	48	F	Dolnośląskie

550.	-	-	12	F	Dolnośląskie
551.	-	-	108	F	Dolnośląskie
552.	-	-	24	F	Dolnośląskie
553.	-	-	6	M	Dolnośląskie
554.	-	-	6	M	Dolnośląskie
555.	-	-	24	F	Dolnośląskie
556.	-	-	72	F	Dolnośląskie
557.	-	-	24	F	Dolnośląskie
558.	-	-	24	F	Dolnośląskie
559.	-	-	24	F	Dolnośląskie
560.	-	-	24	F	Dolnośląskie
561.	-	-	24	F	Dolnośląskie
562.	-	-	24	F	Dolnośląskie
563.	-	-	72	F	Dolnośląskie
564.	-	-	48	F	Dolnośląskie
565.	-	-	132	F	Dolnośląskie
566.	-	-	36	F	Dolnośląskie
567.	-	-	60	F	Dolnośląskie
568.	-	-	60	F	Dolnośląskie
569.	-	-	60	F	Dolnośląskie
570.	-	-	9	F	Dolnośląskie
571.	-	-	168	F	Dolnośląskie
572.	-	-	60	F	Dolnośląskie
573.	-	-	36	F	Dolnośląskie
574.	-	-	120	F	Dolnośląskie
575.	-	-	60	F	Dolnośląskie
576.	-	-	48	F	Dolnośląskie
577.	-	-	36	M	Dolnośląskie
578.	-	-	36	F	Dolnośląskie
579.	-	-	60	F	Dolnośląskie
580.	-	-	24	F	Dolnośląskie
581.	-	-	72	F	Dolnośląskie
582.	-	-	60	F	Dolnośląskie
583.	-	-	48	F	Dolnośląskie
584.	-	-	24	F	Dolnośląskie
585.	-	-	8	F	Dolnośląskie
586.	-	-	8	M	Dolnośląskie
587.	-	-	8	F	Dolnośląskie
588.	-	-	8	M	Dolnośląskie
589.	-	-	8	F	Dolnośląskie
590.	-	-	6	F	Dolnośląskie
591.	-	-	8	F	Dolnośląskie
592.	-	-	8	F	Dolnośląskie
593.	-	-	8	F	Dolnośląskie
594.	-	-	36	F	Dolnośląskie
595.	-	-	9	F	Dolnośląskie

596.	-	-	8	F	Dolnośląskie
597.	-	-	24	F	Dolnośląskie
598.	-	-	96	F	Dolnośląskie
599.	-	-	84	F	Dolnośląskie
600.	-	-	48	F	Dolnośląskie
601.	-	-	132	F	Dolnośląskie
602.	-	-	48	F	Dolnośląskie
603.	-	-	60	F	Dolnośląskie
604.	-	-	72	F	Dolnośląskie
605.	-	-	72	F	Dolnośląskie
606.	-	-	60	F	Dolnośląskie
607.	-	-	48	F	Dolnośląskie
608.	-	-	60	F	Dolnośląskie
609.	-	-	36	F	Dolnośląskie
610.	64	64	84	F	Lubelskie
611.	32	32	60	F	Lubelskie
612.	64	64	72	F	Lubelskie
613.	16	64	72	F	Lubelskie
614.	64	64	48	F	Lubelskie
615.	32	32	72	F	Lubelskie
616.	64	64	48	F	Lubelskie
617.	32	64	24	F	Lubelskie
618.	128	512	60	F	Lubelskie
619.	16	64	108	F	Lubelskie
620.	64	32	48	F	Lubelskie
621.	128	64	84	F	Lubelskie
622.	512	1024	84	F	Lubelskie
623.	64	64	72	F	Lubelskie
624.	128	128	96	F	Lubelskie
625.	64	256	84	F	Lubelskie
626.	256	256	24	F	Lubelskie
627.	256	128	24	F	Lubelskie
628.	32	64	72	F	Lubelskie
629.	32	32	72	F	Lubelskie
630.	32	8	36	F	Lubelskie
631.	16	32	96	F	Lubelskie
632.	32	16	60	F	Lubelskie
633.	64	32	60	F	Lubelskie
634.	256	128	24	F	Lubelskie
635.	128	256	36	F	Lubelskie
636.	512	256	84	F	Lubelskie
637.	64	256	60	F	Lubelskie
638.	64	16	24	F	Lubelskie
639.	128	256	72	F	Lubelskie
640.	64	128	96	F	Lubelskie
641.	64	256	60	F	Lubelskie

642.	64	256	60	F	Lubelskie
643.	128	128	84	F	Lubelskie
644.	128	8	48	F	Lubelskie
645.	128	256	36	F	Lubelskie
646.	128	64	24	F	Lubelskie
647.	32	64	84	F	Lubelskie
648.	64	512	60	F	Lubelskie
649.	128	16	96	F	Lubelskie
650.	16	16	60	F	Lubelskie
651.	128	256	84	M	Lubelskie
652.	16	128	84	M	Lubelskie
653.	32	512	72	F	Lubelskie
654.	-	-	36	F	Lubelskie
655.	-	-	72	F	Lubelskie
656.	-	-	36	F	Lubelskie
657.	-	-	60	F	Lubelskie
658.	-	-	72	F	Lubelskie
659.	-	-	60	F	Lubelskie
660.	-	-	48	F	Lubelskie
661.	-	-	48	F	Lubelskie
662.	-	-	24	F	Lubelskie
663.	-	-	36	F	Lubelskie
664.	-	-	36	F	Lubelskie
665.	-	-	36	F	Lubelskie
666.	-	-	72	F	Lubelskie
667.	-	-	24	F	Lubelskie
668.	-	-	60	F	Lubelskie
669.	-	-	60	F	Lubelskie
670.	-	-	36	F	Lubelskie
671.	-	-	60	F	Lubelskie
672.	-	-	36	F	Lubelskie
673.	-	-	24	F	Lubelskie
674.	-	-	48	F	Lubelskie
675.	-	-	36	F	Lubelskie
676.	-	-	48	F	Lubelskie
677.	-	-	48	F	Lubelskie
678.	-	-	48	F	Lubelskie
679.	-	-	60	F	Lubelskie
680.	-	-	12	F	Lubelskie
681.	-	-	24	F	Lubelskie
682.	-	-	24	F	Lubelskie
683.	-	-	96	F	Lubelskie
684.	-	-	36	F	Lubelskie
685.	-	-	36	F	Lubelskie
686.	-	-	60	F	Lubelskie
687.	-	-	120	M	Lubelskie

688.	-	-	84	F	Lubelskie
689.	-	-	168	M	Lubelskie
690.	-	-	132	F	Lubelskie
691.	-	-	120	F	Lubelskie
692.	-	-	60	F	Lubelskie
693.	-	-	60	M	Lubelskie
694.	-	-	72	M	Lubelskie
695.	-	-	60	F	Lubelskie
696.	-	-	24	F	Lubelskie
697.	-	-	84	M	Lubelskie
698.	-	-	60	F	Lubelskie
699.	-	-	48	F	Lubelskie
700.	-	-	108	M	Lubelskie
701.	-	-	96	F	Lubelskie
702.	-	-	72	F	Lubelskie
703.	-	-	48	F	Lubelskie
704.	-	-	48	F	Lubelskie
705.	-	-	72	M	Lubelskie
706.	-	-	60	F	Lubelskie
707.	-	-	48	F	Lubelskie
708.	-	-	108	F	Lubelskie
709.	-	-	72	F	Lubelskie
710.	-	-	72	F	Lubelskie
711.	-	-	96	F	Lubelskie
712.	-	-	60	F	Lubelskie
713.	-	-	24	F	Lubelskie
714.	-	-	96	F	Lubelskie
715.	-	-	36	F	Podkarpackie
716.	-	-	84	F	Podkarpackie
717.	-	-	36	F	Podkarpackie
718.	-	-	72	F	Podkarpackie
719.	-	-	60	F	Podkarpackie
720.	-	-	60	F	Podkarpackie
721.	-	-	84	F	Podkarpackie
722.	-	-	96	F	Podkarpackie
723.	-	-	36	F	Podkarpackie
724.	-	-	8	F	Podkarpackie
725.	-	-	72	F	Podkarpackie
726.	-	-	36	F	Podkarpackie
727.	-	-	84	F	Podkarpackie
728.	-	-	108	F	Podkarpackie
729.	-	-	84	F	Podkarpackie
730.	-	-	12	F	Podkarpackie
731.	-	-	72	F	Podkarpackie
732.	-	-	60	F	Podkarpackie
733.	-	-	24	F	Podkarpackie

734.	-	-	48	F	Podkarpackie
735.	-	-	168	F	Podkarpackie
736.	-	-	84	F	Podkarpackie
737.	-	-	24	F	Podkarpackie
738.	-	-	24	F	Podkarpackie
739.	-	-	24	F	Podkarpackie
740.	-	-	24	F	Podkarpackie
741.	-	-	60	F	Podkarpackie
742.	-	-	24	F	Podkarpackie
743.	-	-	12	F	Podkarpackie
744.	-	-	24	F	Podkarpackie
745.	-	-	12	F	Podkarpackie
746.	-	-	24	F	Podkarpackie
747.	-	-	60	F	Podkarpackie
748.	-	-	60	F	Podkarpackie
749.	-	-	36	F	Podkarpackie
750.	-	-	144	F	Podkarpackie
751.	-	-	36	F	Podkarpackie
752.	-	-	24	F	Podkarpackie
753.	-	-	120	F	Podkarpackie
754.	-	-	36	F	Podkarpackie
755.	64	64	96	F	Podkarpackie

Age M – age, in months
N/A – not available
F – female
M – male
